# Teenage Neurologic Manifestation of a Complex Brain Malformation Consisting of Gray Matter Heterotopia, Schizencephaly and Absent Septum Pellucidum

**DOI:** 10.5334/jbsr.2577

**Published:** 2021-10-14

**Authors:** Nico Hustings, Marc Lemmerling

**Affiliations:** 1 UZ Leuven, BE; 2 AZ Sint-Lucas, BE

**Keywords:** Congenital brain malformation, Dystonia, Upper motor neuron syndrome

## Abstract

**Teaching point**: Since some minor congenital brain defects manifest long after birth, even in adults they should be kept in the differential of new epileptic seizures or focal neurologic deficits.

## Case

**Figure 1:**
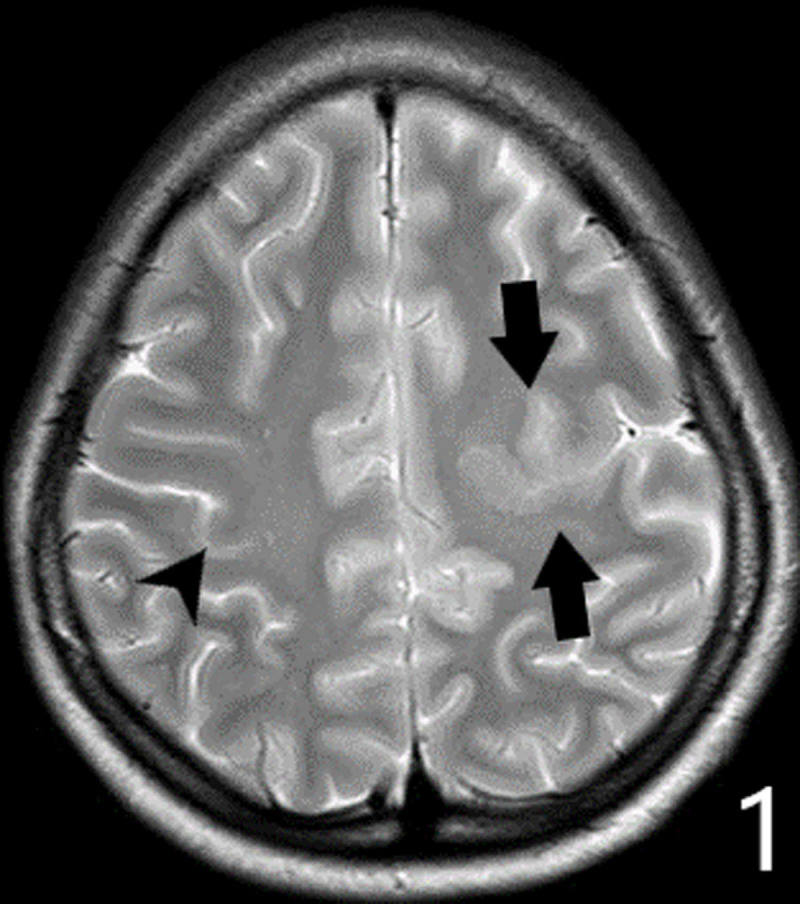


**Figure 2:**
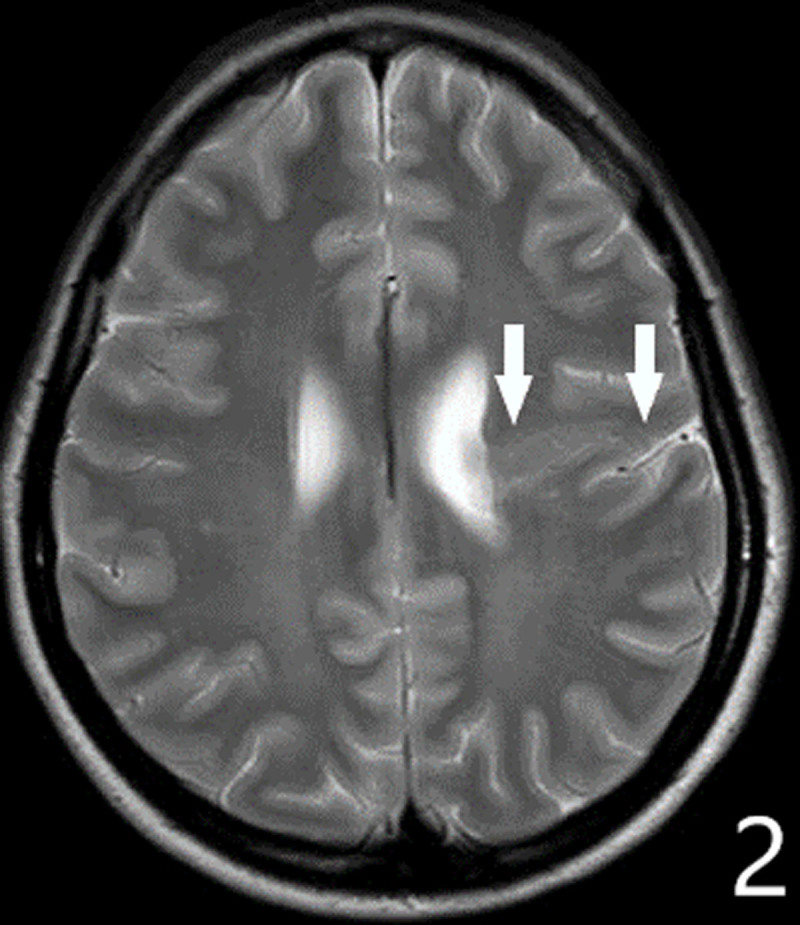


**Figure 3:**
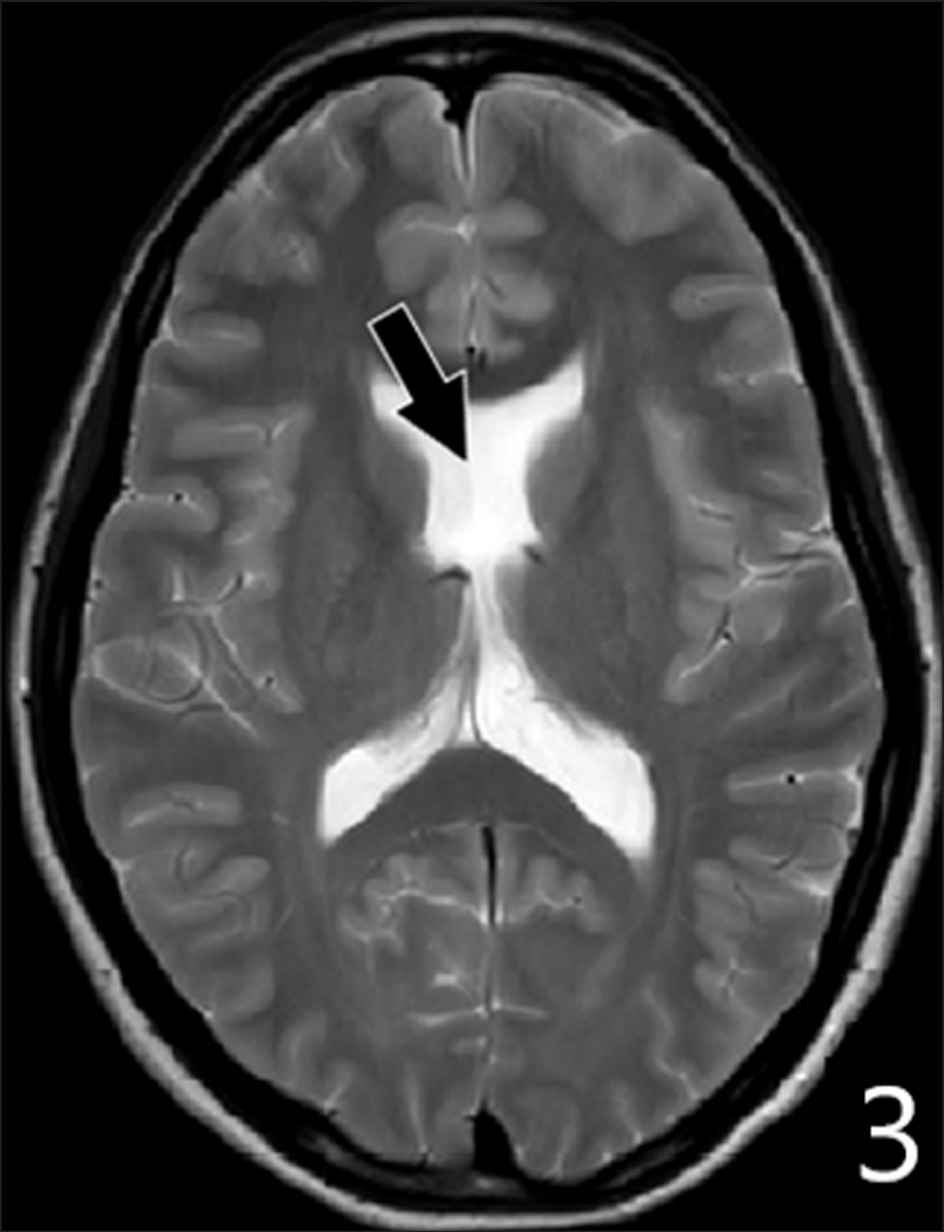


A 16-year-old girl presented with declining motor function of the right hand over the last four years. She complained of reduced force, loss of fine motor skills and episodes of painful muscular cramps. Clinical examination showed athetotic finger movements at rest. Painful contractions were provoked by performing tasks. Muscular atrophy was noted. Finger tapping test confirmed loss of fine motor control and augmented reflexes were found in the arm. The movement disorder was clinically described as a unilateral hand dystonia (with phasic and tonic components) combined with upper motor neuron syndrome (muscle weakness, hyperreflexia and reduced fine motor skills). The muscular atrophy, a sign of lower motor neuron syndrome, was attributed to disuse. Brain MRI revealed multiple congenital abnormalities in the left cerebral hemisphere. ***Figure 1*** shows gray matter heterotopia deforming the left-hand knob (black arrows); compare with the normal right-hand knob (black arrowhead). ***Figure 2*** shows schizencephaly in the left precentral region (white arrows): a gray matter-lined cleft filled with cerebrospinal fluid extending from the ependyma of the lateral ventricle to the subarachnoid space of frontal region. ***Figure 3*** demonstrates an absent septum pellucidum (black arrow).

These congenital malformations in the left hand knob topographically correlated with the dysfunctional right hand. However, it remained remarkable that this congenital defect led to symptoms with late onset and slow progression in this 16-year-old girl. Congenital malformations of the cerebral cortex usually present at birth or in the first decade of life (causing epileptic seizures, focal neurologic deficits or developmental decay). However, as proven in by our case, congenital defects can be associated with normal intelligence and normal neurodevelopment, and some may eventually develop symptoms later in life.

## Comment

Gray matter heterotopia (i.e. ectopic presence of gray matter) is a common malformation of cortical development resulting from the failed neuronal migration away from the embryonic ventricular zone towards the cortex [1]. On MRI, the signal intensities of the nodules are identical to those of normal gray matter. The main differential diagnosis of subependymal heterotopia is tuberous sclerosis, in which the subependymal nodules are calcified on computed tomography and hyperintense on T2-WI. Schizencephaly is a gray matter lined cleft extending from the ependyma to the pia mater, which is a rare anomaly with an estimated incidence of 1.5 per 100.000 live births. Absence of the septum pellucidum is a rare finding, postnatally occurring in 2 per 100.000 individuals. An isolated absence of the septum is very rare, it is typically associated with other intracranial developmental disorders. Therefore, a missing septum pellucidum mandates further investigation to detect and characterize accompanying abnormalities.
